# An Investigation of Natural Background Radiation Levels in Different Locations of Saudi Arabia

**DOI:** 10.7759/cureus.79343

**Published:** 2025-02-20

**Authors:** Oinam Gokulchandra Singh, Ali Aldhebaib, Reem Ibrahim Mohammed Hantol, Sarah Algahtani, Sarah Al Mutairi, Mona Alenazi, Abdulmajeed Alotaibi, Noha Al Thubaity, Fayaz Ul Haq, Alaa Alangary, Winnie Philip, Bader Aldebasi

**Affiliations:** 1 Radiological Sciences, College of Applied Medical Sciences, King Saud Bin Abdulaziz University for Health Sciences, Riyadh, SAU; 2 King Abdullah International Medical Research Center, Ministry of National Guard Health Affairs, Riyadh, SAU; 3 Research Unit, College of Applied Medical Sciences, King Saud Bin Abdulaziz University for Health Sciences, Riyadh, SAU; 4 Research Promotion and Education Section, King Abdullah International Medical Research Center, Riyadh, SAU

**Keywords:** absorbed dose, annual effective dose (aed), background radiation (br), environmental radiation meter, excess lifetime cancer risk (elcr)

## Abstract

Background: Ionizing natural background radiation (BR) has existed on Earth's surface since the beginning of biological life. The daily routine cannot be isolated from radiation exposure, particularly BR. This study aims to investigate natural BR in different areas of Saudi Arabia.

Materials and methods: The study included 10 regions in Saudi Arabia, including the south, north, middle, west, and east of Riyadh, north and south of Jeddah, east and south of Najran, and the Dammam region. The study utilized a portable Environmental Radiation Meter Type GCA-07 series (Digital Geiger counter, images scientific instrument, Inc, Staten Island NY), calibrated by the standard radioactive source of Cs-137 in the National Institute of Standards and Technology (NIST) to investigate BR across various regions. The BR was accessed by measuring the absorbed dose to estimate the annual effective dose (AED) indoors. It involved positioning the detector at least four meters away from any building or wall and maintaining it one meter above the ground surface to assess BR.

Results: The absorbed dose rate of BR results revealed that the highest and lowest were 0.18 mSv/hr and 0.11 mSv/hr in south Jeddah and Dammam. The data indicate that these areas had higher mean indoor AED than the global BR average. Similarly, the excess life cancer risk (ELCR) shows the same results, with the highest and lowest being 3.69 x 10^-3^ and 2.17x10^-3^ in south Jeddah and Dammam. These values are higher than the world global average of ELCR.

Conclusions: The outcome shows that the indoor BR in Saudi Arabia is above the worldwide mean of AED. Similar studies with extensive data collection across all regions of Saudi Arabia may be required to get enough empirical data about natural BR in future research.

## Introduction

Natural background radiation (BR) that is ionizing has been present on Earth's surface since biological life first appeared. However, gamma radiation emits BR. The gamma rays come from higher energy from the atmosphere and isotope decay in the rock. Large concentrations of radium and its decay products are the principal sources of BR [1]. Also, gamma radiation is produced naturally in the air and indoor radon gas [2]. There are three basic categories of BR sources: cosmic, terrestrial, and cosmogenic radiation [1,3]. Cosmic radiation includes secondary highly energetic particles produced by spallation reactions with primary cosmic rays and atmospheric nuclei [4]. However, the routine of daily life cannot be separated from radiation exposure, specifically exposure to natural radiation. For instance, in the earth's crust, there are radioactive elements that lead to human exposure to potassium (40 K), uranium (238U), and thorium (232T). Furthermore, there are radioactive decay products, such as radium (226Ra) and radon (222Rn) [5]. The amount of radiation received by humans is out of control and comes from natural sources in about 82% of cases [1]. Several epidemiological studies have been carried out to assess the risk of cancer incidence in the world's high background natural radiation areas (HBNRAs). The finding of these studies is that not all cancer-related fatalities are attributed to radiation exposure [6]. Nevertheless, there is a paper that reviews the potential contribution of studies of populations living in areas of high natural radiation (HNBR), such as Guarapari, Brazil; Kerala, India; Ramsar, Iran; Yangjiang, and China. The outcomes from this paper provided effective evidence of an association between long-term radiation exposure and disease incidence [7]. Another study conducted on the U.S. population revealed that exposure to high BR shows clear beneficial health effects in humans. These hormetic effects suggest a compelling need to reconsider the linear no-threshold paradigm, particularly within the natural range of low-dose radiation [8].

A similar study measured radioactivity concentrations of 226 Ra, 232Th, and 40K in diﬀerent fertilizers used in Saudi Arabia. The finding from this study is that heavy metals in chemical and organic fertilizers aﬀected human health [9]. Additionally, in the western and southwestern parts of Saudi Arabia, including Makkah, Al Baha, Assir, Jazan, and Najran, it was examined how much gamma radiation was naturally present in the air and how much radon gas was present indoors. In addition, the results state that the indoor gamma radiation dose rate and radon concentration in Riyadh City are within the global average dose of 0.48 mSv as established by various monitoring agencies [10]. The estimate of radiological dangers and the determination of natural radionuclide concentrations are crucial for ensuring public health. Particularly in locations with high radiation levels, this is true. Some operations, like mining and the ore sorting procedure, have the potential to affect activity concentration [11]. The estimated median population attributable risk of lung cancer mortality from residential radon in 2012 across 66 countries with representative national radon surveys was found to be consistent, with 16.5%, 14.4%, and 13.6% for the exposure-age-concentration model (BEIR VI), the Hunter model, and the Kreuzer model, respectively [12]. Another study found that indoor and outdoor radon concentrations in Mamuju, Indonesia could reach several hundred becquerels per cubic meter Bq m^-3^ measuring the radioactive concentration in a medium, far surpassing the levels reported in regions where radon-related lung cancer risks have been identified [13]. The gamma radiation dose rate in outdoor and indoor environments in the Gachine area exceeds the global average dose rate of 0.48 mSv as reported by UNSCEAR [14]. A subsequent study conducted on the Japanese population assessed natural radiation exposure. The estimated arithmetic mean annual effective doses were 0.29 mSv from cosmic rays, 0.33 mSv from terrestrial radiation, 0.59 mSv from radon, and 0.99 mSv from food. Consequently, the total annual dose from natural radiation for the Japanese population was calculated to be 2.2 mSv, closely aligning with the global average of 2.4 mSv [15].

Although radiation benefits people’s lives (e.g., medicine, pharmacy, engineering, agriculture), care must be taken to prevent adverse health effects from radiation exposure. Accordingly, the use of radiation is controlled with reference to the ‘radiation dose,’ which is an index for estimating the level of exposure. Many regions, especially in developing countries and remote areas, lack detailed natural BR measurements. Studies are often focused on specific locations (e.g., urban areas) rather than providing a comprehensive national or global map. In this paper, the main objective is to investigate natural BR across various regions in Saudi Arabia. 

## Materials and methods

Geography of the study area and measurement setup

To gauge BR, the study utilized a portable environmental radiation meter type GCA-07 series (Digital Geiger counter, images Scientific Instrument, Inc, Staten Island NY), calibrated by the standard radioactive source of Cs-137 in the National Institute of Standards and Technology (NIST). The device is applied to detect alpha, beta, gamma, and x-ray radiation; in addition, the sensitivity of the detector for alpha, beta, and gamma is above 3.0MeV, above 50KeV, and above 7Kev, receptively. The study examined 10 regions across Saudi Arabia, including north, east, west, south, and central Riyadh; northern and southern Jeddah; eastern and southern Najran; and the southern Dammam region. Within each designated area, neighborhoods were selected using the city center as a reference point to assess indoor BR levels. The measurements involved holding the detector at least four meters away from any building or wall and maintaining it one meter above the ground surface to assess BR. The absorbed dose rate was calculated using the indoor annual effective dose, considering an exposure duration of one hour for each data point, with a total exposure time of three hours for three readings. The mean of the measurements in each building was computed and considered as the indoor absorbed dose of that building, and the results of this study were compared with world quantities. Specific inclusion and exclusion criteria were implemented to reduce bias.

Inclusion Criteria

Air samples were collected from 10 different geographical regions across Saudi Arabia. The measurement of the activity must be only the occupied area.

Exclusion Criteria

Open space measurement activities were excluded.

AED values and excess risk

The values of the absorbed dose rate were used to calculate the indoor AED rate considering some correction factors; additionally, AED from BR was obtained:

AED_indoor_ (mSv.y-1) = Absorbed dose rate (nGy/h) x T(h) x 0.8 x 0.7 Sv/Gy

Where AED is the annual effective dose (mSv.y-1), and T is the time converter from hour to year (8760h). The AED was calculated using an indoor occupancy factor of 0.8, which represents the fraction of total time an individual spends exposed to a radiation field. The dose conversion coefficient used was 0.7 Sv/Gy to convert the absorbed dose in air to an effective dose in humans. The excess lifetime cancer risk (ELCR) was calculated using the following formula:

 ELCR = AED × DL × RF [16].

Where AED is the annual effective dose, DL is the average lifespan (70 years), and RF is the risk factor (Sv-¹).

Data analysis

The data were transferred to an Excel sheet, and analysis was performed using IBM SPSS Statistics for Windows, Version 29 (Released 2023; IBM Corp., Armonk, New York, United States). Based on variables, a one-way analysis of variance (ANOVA) and repeated measures ANOVA test were conducted to measure the P-value among the readings and areas. The data, expressed as the mean and standard deviation in mSv/h, were confirmed to follow a normal distribution according to the Shapiro-Wilk test.

## Results

**Figure 1:**
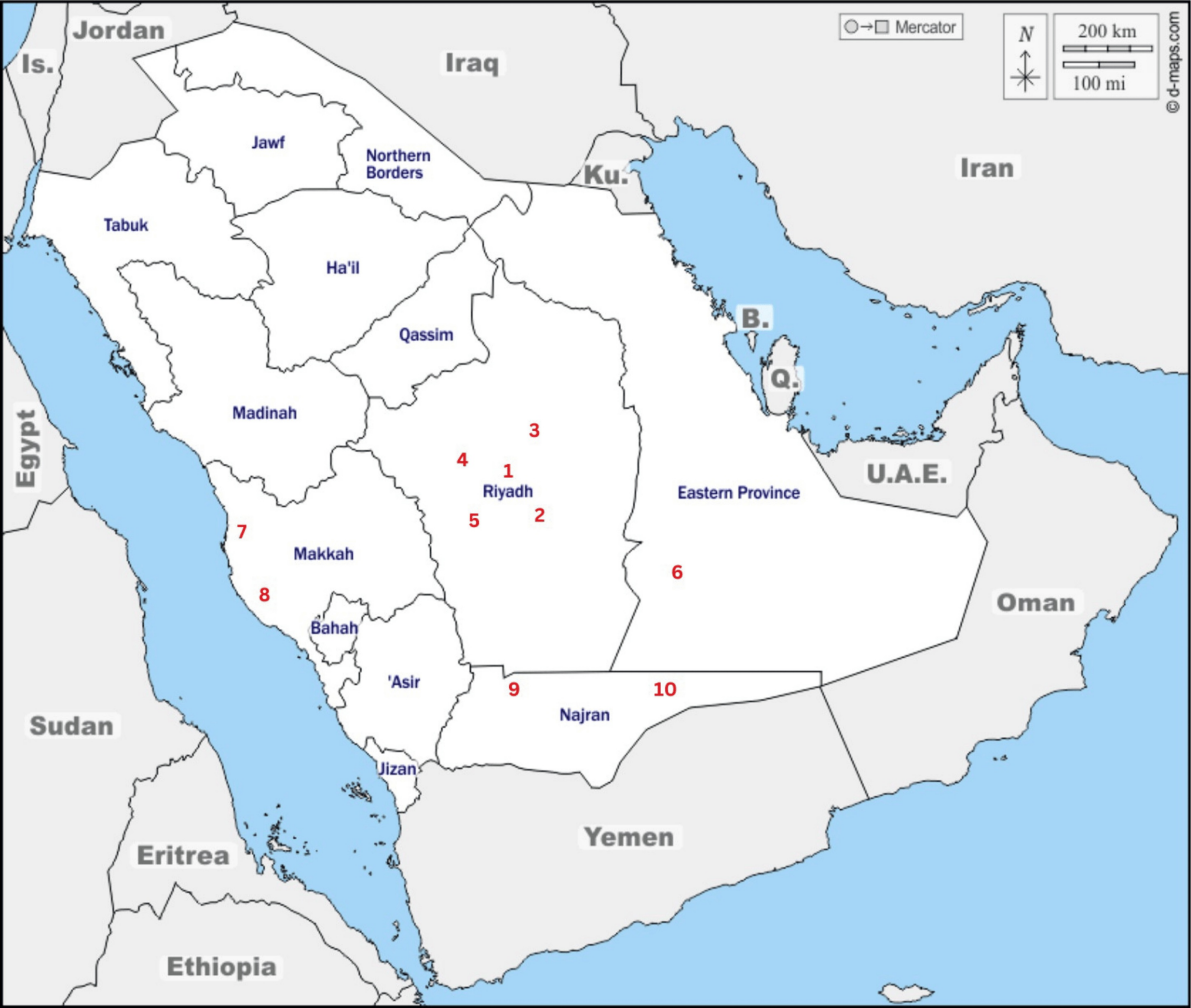
A map of selected areas used in this study 1. Middle of Riyadh, 2. South of Riyadh, 3. East of Riyadh, 4. North of Riyadh, 5. West of Riyadh, 6. Dammam, 7. North of Jeddah, 8. South of Jeddah, 9. North Najran, 10. East Najran The image is modifiable and free to use and publish under a Creative Common License [17].

The BR levels were measured to establish the AED indoors in different areas of Saudi Arabia. The BR measurements from 10 areas were performed using the CCA-07 series Geiger Counter, showing three readings per area. The results include the mean and standard deviation (SD) of the measurements. The mean absorbed dose rate from BR ranged from the lowest of 0.11 mSv in Dammam to the highest of 0.18 mSv in South Jeddah, with the latter also exhibiting the highest variability (SD of 0.11). In Riyadh, the readings varied from 0.11 mSv in East Riyadh to 0.14 mSv in Middle Riyadh, with South Riyadh showing a lower mean of 0.12 mSv and minimal variability (SD of 0.0051). Other areas like North Jeddah, South Najran, and East Najran had mean values between 0.13 mSv and 0.15 mSv, with moderate standard deviations ranging from 0.017 to 0.034. Overall, the radiation levels varied across regions, with South Jeddah showing the most fluctuation. Five areas within Riyadh, including south, north, middle, west, and east, were determined for measurement. Additionally, two areas in the north and south of Jeddah, followed by the north and east of Najran region and Dammam region located in the eastern part of Saudi Arabia, were also included as illustrated in Figure 1. The mean and standard deviation are expressed in mSv/h; data are normally distributed according to the Shapiro-Wilk test. The highest value is 0.188±0.113 in south Jeddah, and the lowest is 0.111±0.0127 in Dammam (Table 1). The relatively low standard deviation values for each of the ten areas indicate that the distribution of the absorbed dose rate in the study area is uniform. This distribution also reflects the homogeneity of the geology within the research area. Increasing the mean value indicates increased adsorbed dose rates, which also increases effective dose rates and ELCR.

**Table 1:** Different areas included in the study with three readings measured using GCA-07 series Geiger Counter; additionally, the table shows the mean and standard deviation (SD) for each area GCA-07 series Geiger Counter: Images Scientific Instrument, Inc, Staten Island NY

	Area	First Reading (mSv)	Second Reading (mSv)	Third Reading (mSv)	Mean ±SD
1	South Riyadh	0.11	0.12	0.12	0.12 ±0.0051
2	North Riyadh	0.12	0.15	0.12	0.13±0.0144
3	Middle Riyadh	0.13	0.14	0.16	0.14±0.0172
4	West Riyadh	0.15	0.13	0.15	0.14±0.0144
5	East Riyadh	0.11	0.12	0.10	0.11±0.0085
6	Dammam	0.1	0.12	0.10	0.11±0.0127
7	South Jeddah	0.1	0.31	0.15	0.18±0.1130
8	North Jeddah	0.15	0.09	0.15	0.13±0.0340
9	South Najran	0.14	0.13	0.17	0.14±0.0223
10	East Najran	0.15	0.13	0.16	0.15±0.0172
ANOVA test/SD: Standard Deviation, mSv: millisieverts, ELCR: Excess Lifetime Cancer Risk					

As shown in Table 2, South Jeddah recorded the highest AED indoor, while Dammam had the lowest AED indoor. However, there is no significant difference between the two values as the exposure in the two areas is nearly similar. Based on variables, an ANOVA test was conducted to measure the P-value across the readings and areas. Firstly, we compared the first, second, and third readings across all areas. The result shows no significant difference across all areas with the P-value equal to 0.129, as shown in Table 3.

**Table 2:** Results of calculated absorbed dose rates, effective dose rates, and ELCR of different areas in the study ELCR: Excess Lifetime Cancer Risk, mSv: millisieverts

Area	Absorbed Dose Rates (mSv/hr)	Effective Dose Rates (mSV/y)	ELCR
South Riyadh	0.12	0.59	2.38
North Riyadh	0.13	0.65	2.60
Middle Riyadh	0.14	0.71	2.87
West Riyadh	0.14	0.73	2.92
East Riyadh	0.11	0.57	2.27
Dammam	0.11	0.54	2.17
South Jeddah	0.18	0.92	3.69
North Jeddah	0.13	0.63	2.55
South Najran	0.14	0.73	2.92
East Najran	0.15	0.74	2.98

**Table 3:** Comparison of three reading using Portable Environmental Meter Type GCA-07 Series

	Mean±SD			
Variable	First Reading	Second Reading	Third Reading	P-Value
Absorbed Dose	0.12 ± 0.02	0.14± 0.06	0.14 ± 0.02	0.12
ANOVA test/SD: Standard Deviation, ELCR: Excess Lifetime Cancer Risk				

Similarly, variation in absorbed dose rates, effective dose rates, and ELCR across the main four regions, Riyadh, Jeddah, Najran, and Dammam, was analyzed using the ANOVA test. The results show no significant differences in the absorbed dose, effective dose, and ELCR with a P-value of 0.286 for all the variables, as shown in Table 4.

**Table 4:** Comparison of doses between the areas ANNOVA test used/SD: Standard Deviation, ELCR: Excess Lifetime Cancer Risk

Variables	Mean±SD				P-Value
	Riyadh	Jeddah	Dammam	Najran	
Absorbed Dose Rates (Gy)	0.13±0.014	0.16±0.042	0.11±0.000	0.151±0.002	0.28
Effective Dose Rates (mSv)	0.65±0.070	0.78±0.20	0.54±0.000	0.741±0.009	0.28
ELCR	2.6±0.281	3.12±0.806	2.17±0.000	2.956±0.037	0.28

The absorbed dose rate of BR showed that the highest and lowest rates were 0.18 mSv/hr in South Jeddah and 0.11 mSv/hr in Dammam, respectively. The data indicate that these areas had a higher mean indoor AED compared to the global BR average of 0.48 mSv. Similarly, the ELCR exhibited similar findings, with values of 3.69 x 10⁻³ in South Jeddah and 2.17 x 10⁻³ in Dammam, both higher than the global average ELCR of 0.29 x 10⁻³.

## Discussion

According to the UNSCEAR 2000 study, there can be fluctuations in natural BR exposure levels throughout the world, but these changes often occur within a range that, in normal environmental circumstances, does not represent a major risk to human health. The results from Riyadh, Jeddah, Dammam, and Najran are all within these predicted ranges, providing more evidence that the radiation doses in these Saudi regions are safe and comparable [16]. This study's effective dose rates are similar to prior studies, such as Harb's (2008) study on natural radioactivity and gamma radiation exposure near the coastal Red Sea in Egypt [18], which found an effective dose rate of 2.0 ± 1.5 mSv. Harb's findings highlight the heterogeneity of natural BR levels, which may be impacted by geography and environmental variables. While the effective dose rates in the current study are not expressly stated, they appear to be consistent with Harb's reported natural fluctuation, indicating that radiation levels in Riyadh, Jeddah, Dammam, and Najran are within the anticipated range for such locations. When two separate studies' conclusions are compared, the difference in natural radiation levels between places becomes clear. A 2009 survey in Yalvaç County found low natural BR levels of around 0.3 µSv/h throughout midday and overnight periods. Another study revealed that radon exposure can cause specifically alpha particles-induced lung cancer and DNA damage. BR has been a concern to measure by researchers internationally [19,20]. 

A large number of researchers have looked into the mean activity concentrations of radioactive elements, including 40K, 238U, and 232Th, in the earth's crust using BR quantities. In this study, the BR was measured corresponding to indoor AED by using the portable Environmental Radiation Meter type GCA-07 series (Digital Geiger counter, Images Scientific Instrument, Inc, Staten Island NY), calibrated by the standard radioactive source of Cs-137 in National Institute of Standards and Technology (NIST). The result shows that the AED _indoor_, which has been measured in five areas within Riyadh, north and south of Jeddah, Dammam, and north and east of Najran, shows that the doses are relatively close to each other, with the highest value found in south Jeddah and the lowest in Dammam. Additionally, the ELCR shows the same value as the highest and lowest values in south Jeddah and Dammam, respectively.

In contrast, a more recent 2015 study done in Ramsar investigated external radiation levels both indoors and outdoors, with a particular emphasis on Ramsar's HBNRAs and normal background radiation areas [7]. The findings demonstrated a large variance in yearly effective doses to the population, with HBNRAs registering doses ranging from 0.6 to 131 mSv, averaging six mSv per year, and NBRAs registering doses ranging from 0.6 to 1.5 mSv, averaging 0.7 mSv per year. 

This significant variation emphasizes the impact of geography and environmental factors on natural radiation exposure rates. Similarly, our research on ELCR in diverse Saudi regions generated useful results. Notably, the p-value for AED in Riyadh, Jeddah, Dammam, and Najran was calculated to be 0.286, showing a significant link that warrants further examination.

Furthermore, our findings revealed a trend in which the ELCR in these regions exceeded that of the western region, stressing the necessity for targeted methods to overcome regional differences. Overall, these findings highlight the significance of location-specific analysis in comprehending and managing complex socioeconomic and environmental concerns.

A 2022 study conducted in the western region of Saudi Arabia found that children's annual effective doses ranged from 0.58 to 5.78 mSv, with an average value of 2.12 mSv [21]. For adults, the doses ranged from 0.61 to 4.28 mSv, with an average of 1.86 mSv. In comparison, the AED p-value in Riyadh, Jeddah, Najran, and Dammam was 0. 286. It was also found that the ELCR in other regions of Saudi Arabia was higher than that in the western region. Another study at Umm Al-Qura University in Saudi Arabia measured BR at 0.012 mSv, which is lower than the AED in this study [22].

‏The strength of the study lies in the meticulous approach to data collection, where the area was measured three times for more consistency and accuracy. On the other hand, the limitations of this study are related to climatic factors and geographical characteristics of the regions that had an impact on measurement outcomes. It is important to acknowledge these limitations when interpreting study results to ensure a comprehensive understanding of the research findings. Other monitoring devices, such as thermoluminescent dosimeters, could be used to verify their accuracy and reliability through comparative analysis. Finally, conducting multiple studies to evaluate natural BR in Saudi Arabia is recommended.

## Conclusions

The investigated indoor AED in selected locations of Saudi Arabia was above the global BR average. Therefore, the advancement of biological inquiries, specifically in the domains of cancer incidence and impact of radiation hematology, will potentially lead to negligibility. On the other hand, this research result information will guide for formulation and implementation of regional surveys dedicated to investigating natural BR in Saudi Arabia. Similar studies with extensive coverage of data collection across all the regions of Saudi Arabia may be required in order to get enough empirical data about the natural BR for future research.
